# Motor, Psychiatric and Fatigue Features Associated with Nutritional Status and Its Effects on Quality of Life in Parkinson’s Disease Patients

**DOI:** 10.1371/journal.pone.0091153

**Published:** 2014-03-07

**Authors:** Seyed-Mohammad Fereshtehnejad, Ladan Ghazi, Mahdiyeh Shafieesabet, Gholam Ali Shahidi, Ahmad Delbari, Johan Lökk

**Affiliations:** 1 Division of Clinical Geriatrics, Department of Neurobiology, Care Sciences, and Society (NVS), Karolinska Institutet, Stockholm, Sweden; 2 Firoozgar Clinical Research Development Center (FCRDC), Firoozgar Hospital, Iran University of Medical Sciences, Tehran, Iran; 3 Department of Biosciences and Nutrition, Karolinska Institutet, Stockholm, Sweden; 4 Medical Students Research Committee (MSRC), Faculty of Medicine, Iran University of Medical Sciences, Tehran, Iran; 5 Movement Disorders Clinic, Department of Neurology, Faculty of Medicine, Iran University of Medical Sciences, Tehran, Iran; 6 Aging Research Center, Sabzevar University of Medical Sciences, Sabzevar, Khorasan, Iran; 7 Department of Geriatric Medicine, Karolinska University Hospital, Stockholm, Sweden; Philadelphia VA Medical Center, United States of America

## Abstract

**Objectives:**

Parkinson’s disease (PD) patients are more likely to develop impaired nutritional status because of the symptoms, medications and complications of the disease. However, little is known about the determinants and consequences of malnutrition in PD. This study aimed to investigate the association of motor, psychiatric and fatigue features with nutritional status as well as the effects of malnutrition on different aspects of quality of life (QoL) in PD patients.

**Methods:**

One hundred and fifty patients with idiopathic PD (IPD) were recruited in this study. A demographic checklist, the Unified Parkinson’s Disease Rating Scale (UPDRS), the Hospital Anxiety and Depression Scale (HADS) and the Fatigue Severity Scale (FSS) were completed through face-to-face interviews and clinical examinations. The health-related QoL (HRQoL) was also evaluated by means of the Parkinson’s Disease Questionnaire (PDQ-39). For evaluation of nutritional status, the Mini Nutritional Assessment (MNA) questionnaire was applied together with anthropometric measurements.

**Results:**

Thirty seven (25.3%) patients were at risk of malnutrition and another 3 (2.1%) were malnourished. The total score of the UPDRS scale (r = −0.613, *P*<0.001) and PD duration (r = −0.284, *P* = 0.002) had a significant inverse correlation with the total MNA score. The median score of the Hoehn and Yahr stage was significantly higher in PD patients with abnormal nutritional status [2.5 vs. 2.0; *P*<0.001]. More severe anxiety [8.8 vs. 5.9; *P* = 0.002], depression [9.0 vs. 3.6; *P*<0.001] and fatigue [5.4 vs. 4.2; *P*<0.001] were observed in PD patients with abnormal nutritional status. Except for stigma, all other domains of the PDQ-39 were significantly correlated with the total score of the MNA.

**Conclusion:**

Our study demonstrates that disease duration, severity of motor and psychiatric symptoms (depression, anxiety) and fatigue are associated with nutritional status in PD. Different aspects of the HRQoL were affected by patients’ nutritional status especially the emotional well-being and mobility domains.

## Introduction

Nutritional status is an important contributor to quality of life (QoL) and general condition of daily living in the elderly [1], [2]. In theory, Parkinson’s disease (PD) patients are susceptible to impaired nutritional status because of different motor and non-motor symptoms, including psychiatric features and fatigue [3]. Moreover, pharmacological treatment administered in PD can influence nutritional status through the drugs themselves and their side effects such as nausea, vomiting and weight loss [4].

Malnutrition can influence immune system, functional status [5], [6], some complications of the disease such as constipation [7] and potentially increases the likelihood of falling [8]. On the other hand, dysphagia increases the risk of malnutrition [9] in PD patients. Despite its important role, nutritional status does not receive the necessary attention in the management of PD and most of the time it is ignored.

So far, there have been a number of studies that have evaluated weight change and/or body mass index (BMI) in PD. However, only a few of them have focused on general nutritional status using validated tools such as the Subjective Global Assessment (SGA) [4], [10] and the mini nutritional assessment (MNA) [11], [12], [13]. Although a few reports used the MNA in PD population, there are still not enough reports to broadly evaluate the relationship between different features of the disease and nutritional status, as well as its effects on the health-related QoL in PD patients. Therefore, our study aimed to investigate the association of motor, psychiatric and fatigue features with nutritional status in PD using the MNA as a comprehensive instrument. In addition, we also evaluated the effects of abnormal nutritional status on different domains of QoL in a population of PD patients.

## Subjects and Methods

One hundred and fifty consecutive patients with idiopathic Parkinson’s disease (IPD) from a single referral Movement Disorders Clinic in Tehran, Iran, were recruited between October 2011 and December 2012. This was a collaborative project between the Iran University of Medical Sciences (Tehran, Iran) and the Karolinska Institutet (Stockholm, Sweden).

### Ethics Statement

The ethics committee of the Firoozgar Clinical Research Development Center (FCRDC) (affiliated with Iran University of Medical Sciences) approved the study protocol. All of the collected data were stored and treated according to the ethical guidelines of medical research. Prior to the launch of the study, all patients were informed about the aims and procedures. All participants provided their verbal informed consent to participate in this study. As the project was designed as an observational research, the verbal form of consent was approved by the aforementioned ethics committee. If any patient did not agree to enroll into the study, no extra evaluation was performed in addition to his/her routine work-up in the clinic and the research checklist was left blank in the documentation procedure. Moreover, participation in this study was voluntary and the patients were free to withdraw from the project whenever they wanted. Furthermore, the identity of research participants was protected, since the data files were anonymous and all of the names were omitted.

### Patient Recruitment

Recruited patients fulfilled the United Kingdom Brain Bank criteria for the diagnosis of IPD [14], which was assessed by the same neurologist who had specialized in movement disorders for all of them. All of the IPD patients who were eligible for this study were required to be 30 years or older with motor disability in the mild to moderate severity range according to the Hoehn and Yahr staging criteria [15]. Patients with moderate to severe dementia [mini-mental state examination (MMSE)<24] [16] were excluded from the study, as were those who were following special diets or suffering from other diseases considerably influencing nutritional state. Also, any patient with atypical Parkinsonism, including multiple system atrophy (MSA), progressive supranuclear palsy (PSP), and vascular or drug-induced Parkinsonism were not eligible.

### Assessments

Data collection was performed through face-to-face interviews with the patients and, whenever possible, their caregivers, along with clinical examinations by means of a checklist and questionnaires. The demographic checklist consisted of baseline variables (age and sex), educational status, co-morbidities, duration of PD (time passed from diagnosis) and history of levodopa administration. These data were collected based on both participants’ self-reports and their medical records at the referral centre. Clinical characteristics of PD were assessed using the Unified Parkinson’s Disease Rating Scale (UPDRS) [17], Hoehn and Yahr stage [15] and Schwab and England activity of daily living (ADL) scale [18]. The Hospital Anxiety and Depression Scale (HADS) [19] questionnaire was used to focus on aspects of anxiety and depression and the Fatigue Severity Scale (FSS) [20] was used for fatigue measurement. Moreover, the health-related quality of life (HRQoL) was also evaluated by means of the Parkinson’s disease quality of life questionnaire (PDQ-39) [21]. In order to assess the nutritional status, the MNA questionnaire was applied together with anthropometric measurements.

All of the assessments were done when the patients were in the “On” state. A movement disorder specialist performed all of the physical examinations and a team of interviewers consisting of trained medical students and general physicians completed the interviews to complete the questionnaires.

### Scales and Questionnaires

#### Unified Parkinson’s Disease Rating Scale (UPDRS)

UPDRS is the most commonly used scale in the clinical study of PD [22], and is used to assess the severity of PD in different aspects including non-motor symptoms (part I), activities of daily living (ADL) (part II), motor examination (part III) and drug complications (part IV). The UPDRS is scored from a total of 147 points where higher scores reflect worsening disability [17].

#### Hoehn and Yahr Stage

The Hoehn and Yahr stage is a widely used clinical rating scale, supplanted by the UPDRS, which defines broad categories of motor function in PD. It evaluates the severity of PD based on functional disability and clinical findings. It contains five stages, where 0 indicates no visible symptoms of PD, and 5 shows symptoms on both sides of the body, identifying those PD patients who are unable to walk. Therefore, a higher stage shows greater levels of functional disability [15].

#### Schwab and England Activities of Daily Living (ADL) scale

The Schwab and England ADL scale is a global scale that is used for assessing a PD patient’s ability to perform daily activities in terms of speed and independence through a percentage figure, where 100% indicates total independence, and 0% indicates a state of complete dependence, which is seen in bed-ridden individuals. Therefore, higher scores show greater level of independence [18].

#### 39-item PD questionnaire (PDQ-39)

The PDQ-39 is the most commonly used instrument for measuring health-related quality of life in PD patients. It has been developed to assess treatments and interventions that may benefit PD patients. In its long format, it contains 39 items assessing eight aspects of QoL in PD patients: mobility, ADL, emotional well-being, stigma, social support, cognitions, communication and bodily discomfort. All questions on the PDQ-39 are coded in a Likert-scale from 0–4, where 0 =  never, 1 =  occasionally, 2 =  sometimes, 3 =  often and 4 =  always. The maximum score of 100 on the PDQ-39 scale represents the worst condition, with a score of zero representing the best level of QoL in PD patients [21]. In this study, we used the Persian-translated version of the PDQ39 questionnaire, which has been previously shown to have a high reliability with a Cronbach’s alpha coefficient of 0.93 for the total summary index [23].

#### Hospital Anxiety and Depression Score (HADS)

The HADS is a screening tool that was designed to assess the levels of anxiety and depression in a non-psychiatric population attending medical clinics. It is comprised of 14 questions divided into two sections; seven questions are related to anxiety and the other seven focus on depression. Each questionnaire is worth 0–21 points, providing separate scores for either the anxiety or depression sub-scales. Responses are determined or calculated by adding up the sum of 0–3 scores for each question, where 0 =  not at all, and 3 =  very often indeed [19]. The Cronbach’s alpha coefficient has been reported as 0.78 for anxiety and 0.86 for the depression sub-scale of the Persian-version of the HADS questionnaire [24] used in our study.

#### Fatigue Severity Scale (FSS)

The FSS is an instrument that assesses the physical aspects of fatigue and their impact on the patient’s daily function in a variety of medical and neurologic disorders. It evaluates the impact of fatigue on motivation, exercise, physical functioning, and carrying out duties and responsibilities, as well as interfering with work, family, or social life. It contains nine questions, with a maximum score of 36 points. We asked each patient to rate the level of fatigue during the past week with scores from 1 to 7 for each statement. Lower values indicate strong disagreement with the statement, whereas higher values indicate strong agreement [20]. A total score is obtained as the average of all of the item-specific scores where higher scores correspond with more severe fatigue. The FSS questionnaire was previously translated into the Persian language and showed acceptable validity and reliability [25]. In this project, we used this Persian-translated version of the FSS questionnaire in our sample of IPD patients.

#### Mini Nutritional Assessment (MNA)

The MNA is a rapid nutrition-assessment tool that can be used for the elderly in order to identify the risk of malnutrition. Moreover, its usefulness extends as a comprehensive geriatric assessment tool that can help in identifying patients who may benefit from early intervention [26]. It has also been seen as a combined screening and assessment tool [27]. The MNA questionnaire consists of brief questions and simple measurements that can be completed in about 10–15 minutes [26]. The full format of the MNA includes 18 items grouped into two sub-sections: six screening questions in the first section and 12 assessment questions in the second section. The MNA scale includes body mass index (BMI), weight loss, arm and calf circumference, appetite, medication, general and cognitive health, dietary matters, autonomy of feeding, and self-perception of health and nutrition, as well as a subjective judgment of malnutrition. The maximum score in the MNA questionnaire is 30 points, where 16 points are obtained by screening questions and 14 points are gained through assessment questions. A total score of less than 17 points indicates “*malnutrition*”, scores of 17–23.5 points signify cases that are “*at risk for malnutrition*”, while scores equal to or more than 24 points represent “*normal nutritional status”* [28]. In this study, we have used the full format of the Persian-translated MNA provided by the *Nestlé* Nutrition Institutet [29] and the total score is reported. Moreover, according to the small sample size in subgroup comparisons, all of the patients with a total MNA score of <24 are mentioned as “*abnormal nutritional status*”, including both malnourished and at-risk patients.

#### Anthropometric Measurements

As a part of the MNA questionnaire, each participant underwent anthropometric measurements including mid arm circumference (MAC), calf circumference (CC), weight and height. Body weight was recorded in a standardised manner using calibrated floor scales while the subjects wore light clothing with no shoes or coats. For all patients, the weight measurement was performed between 3 p.m. and 5 p.m. when they were supposed to have left at least 2 hours since their lunch and had not yet had their dinner. The standing height was measured using a stadiometer at head level, with the subject’s bare feet close together, standing erect and looking straight ahead. Of note, there was no case of considerable stooped posture for any height adjustment. Body mass index was calculated as body weight (*kg*) divided by the square value of height (*m^2^*). For the MAC, the examiner marked the mid-point between the acromial surface of the scapula (bony protrusion surface of upper shoulder) and the olecranon process of the elbow (bony point of the elbow) on the back of the arm, while the subject was asked to hold the forearm in a horizontal position with their palm up. Thereafter, the MAC was measured with a flexible inextensible tape that was applied snugly around the maximum girth of the proximal part of forearm while the subject’s arm was hanging down freely along their trunk at their sides. For the CC, the maximal circumference between the ankle and the knee was measured with a flexible tape that was applied horizontally around the maximum girth of the calf, while the subject was standing with their weight evenly distributed on both feet [30]. In order to accurately measure the MAC and CC, we asked the participants to roll up their trouser leg or sleeve to uncover their calf or arm, respectively.

### Statistical Analysis

All of the data obtained from the checklists and questionnaires were entered into SPSS software version 20 (IBM; Chicago, IL, USA). Numerical variables are described using the mean and standard deviation (SD), whereas, the discrete values of the Hoehn and Yahr stage are presented as the median and interquartile range (IQR). The relative frequency percentage is also used to describe the nominal or categorical variables. The Kolmogorov-Smirnov (K-S) test was used to check the normality of the distribution of the total MNA score, which was shown to be skewed and non-normal (K-S test *P* = 0.003). The univariate relationship between the total MNA score and those obtained from other scales and questionnaires was assessed by means of the Spearman correlation test. In subgroup analysis, the independent samples *T* test was performed to compare the mean scores of different questionnaires between the IPD patients with normal versus abnormal nutritional status. Moreover, the Mann-Whitney *U* test was used to compare the mean of the total MNA score between female and male patients. It must be noted that according to the rather small sample size in subgroup comparisons, the two subgroups of patients with “*malnutrition*” and “at *risk of malnutrition*” were merged together in one group called “*abnormal nutritional status*”.

Further multivariate analysis was performed using a stepwise linear regression model in order to evaluate the factors independently related to the total score of the MNA questionnaire among IPD patients. In addition, binary logistic regression analysis was performed using a forward conditional model to identify the factors that could independently discriminate the IPD patients with abnormal nutritional status. In both regression models, age at the time of diagnosis, sex, weight-adjusted daily levodopa dosage, the score of each part of the UPDRS scale, total UPDRS score, Hoehn and Yahr stage, Schwab and England ADL score, anxiety, depression and fatigue scores were defined as the predictor list. A two-tailed *P*-value of less than 0.05 was considered to show a statistically significant difference or correlation in all analytical procedures.

## Results

### Baseline and Anthropometric Characteristics

In total, 150 PD patients were recruited into this study consisting of 103 (68.7%) males and 47 (31.3%) females with a mean age of 60.8 (SD = 10.8) *yrs,* ranging between 32 and 84 years, and a mean disease duration of 6.8 (SD = 5.3) *yrs*. Table 1 summarises information on the baseline, anthropometric and clinical characteristics of the recruited PD patients. The mean of the total UPDRS score was 31.7 (SD = 17.2) ranging from 6 to 88. The median score of Hoehn and Yahr staging was 2.0 (IQR = 1.5) and the mean percentage of the Schwab and England ADL scale was 81.7% (SD = 16.7).

**Table 1:** Baseline, anthropometric and clinical characteristics of the recruited Parkinson’s disease patients (n = 150).

Characteristics	Value
**Age** *(yr)*-Mean (SD)	60.8 (10.8)
**Gender** *NO.(%)*	
Female	47 (31.3)
Male	103 (68.7)
**Level of Education** *NO.(%)*	
Illiterate	15 (10.1)
Primary and/or Secondary	36 (24.3)
High School/Diploma	41 (27.8)
College and/or University	56 (37.8)
**Duration of Disease** *(yr)* **-**Mean (SD)	6.8 (5.3)
**Co-morbidities** *NO.(%)*	
Depression	36 (24.5)
Hypertension	24 (16.2)
Cardiovascular Disease	23 (15.8)
Osteoarthritis	19 (13.0)
Diabetes	18 (12.3)
**UPDRS Score-**Mean (SD)	
Part I-*mental*	2.1 (2.3)
Part II-*ADL*	11.2 (6.9)
Part III-*motor*	15.3 (8.8)
Part IV-*complications*	3.5 (2.8)
Total	31.7 (17.2)
**Hoehn & Yahr Stage** *-*Median (IQR)	2.0 (1.5)
**Schwab and England Activities of Daily Living Score** *(%)* **-**Mean (SD)	81.7 (16.7)
**Daily levodopa dose-**Mean (SD)	
Crude *(mg)*	812 (490)
Weight-adjusted *(mg/kg)*	11.75 (7.72)
**Anthropometric Measurements-**Mean (SD)	
Weight *(kg)*	71.8 (13.6)
Height *(cm)*	166.7 (8.8)
Body Mass Index (BMI) (*kg/m^2^)*	25.8 (4.2)
Mid-Arm Circumference (MAC) *(cm)*	28.2 (4.9)
Calf Circumference (CC) *(cm)*	34.9 (3.8)

Table 2 shows the descriptive results for nutritional status (MNA), disease-related quality of life (PDQ-39), and severity of anxiety, depression (HADS) and fatigue (FSS) in PD patients. The highest scores (worst conditions) of PDQ-39 were observed in emotional well-being [27.7 (SD = 22.7)] and mobility [26.8 (SD = 25.1)] domains. The mean of the total MNA score was 25.1 (SD = 3.3) and regarding the cut-off points, 37 (25.3%) PD patients were at risk of malnutrition and another 3 (2.1%) cases were already malnourished.

**Table 2:** Descriptive characteristics of nutritional status (MNA), disease-related quality of life (PDQ-39), anxiety, depression (HADS) and fatigue (FSS) in Parkinson’s disease (PD) patients.

Questionnaire/Domain	Mean (SD)	Range
**MNA** (Total)	25.1 (3.3)	6–30
**PDQ-39** *domains*		
Mobility	26.8 (25.1)	0–100
Activities of daily living (ADL)	23.4 (22.9)	0–100
Emotional well-being	27.7 (22.7)	0–100
Stigma	21.6 (25.1)	0–100
Social support	7.1 (13.3)	0–75
Cognitive impairment (Cognition)	16.6 (18.3)	0–81
Communication	14.2 (17.9)	0–83
Bodily discomfort	20.9 (20.8)	0–75
**HADS** *domains*		
Anxiety	6.8 (5.1)	0–20
Depression	5.1 (4.5)	0–17
**FSS**	4.5 (1.9)	1–7

### Univariate Correlations with the MNA Score

Table 3 summarises the univariate correlation coefficients between the scores of different motor, psychiatric, fatigue and quality of life (PDQ-39) scales and nutritional status (MNA). Except for stigma, all other domains of PDQ-39 were significantly and inversely correlated with the total score of the MNA questionnaire (all *P*<0.05). The mobility domain of the disease-related QoL had the largest inverse correlation with the total MNA score (Spearman r = −0.590, *P*<0.001), and the emotional well-being domain was the second most important related factor to the total MNA score (Spearman r = −0.461, *P*<0.001) in the population of recruited PD patients.

**Table 3:** Pearson correlation between the scores of different motor, psychiatric, fatigue and quality of life (PDQ-39) scales and nutritional status (MNA) in Parkinson’s disease (PD) patients.

Scale	Domains	Correlation Index	MNA (Total)
PDQ39	**Mobility**	*Spearman R*	−.590**
		*P-value*	<.001
	**Activities of daily living (ADL)**	*Spearman R*	−.450**
		*P-value*	<.001
	**Emotional well-being**	*Spearman R*	−.461**
		*P-value*	<.001
	**Stigma**	*Spearman R*	−.027
		*P-value*	.744
	**Social support**	*Spearman R*	−.246**
		*P-value*	.005
	**Cognitive impairment (Cognition)**	*Spearman R*	−.320**
		*P-value*	<.001
	**Communication**	*Spearman R*	−.414**
		*P-value*	<.001
	**Bodily discomfort**	*Spearman R*	−.451**
		*P-value*	<.001
Disease Severity	**UPDRS** Part I-*mental*	*Spearman R*	−.503**
		*P-value*	<.001
	**UPDRS** Part II-ADL	*Spearman R*	−.518**
		*P-value*	<.001
	**UPDRS** Part III-motor	*Spearman R*	−.473**
		*P-value*	<.001
	**UPDRS** Part IV-complications	*Spearman R*	−.336**
		*P-value*	<.001
	**UPDRS** Total	*Spearman R*	−.613**
		*P-value*	<.001
	**Hoehn & Yahr stage**	*Spearman R*	−.414**
		*P-value*	<.001
	**Schwab & England stage**	*Spearman R*	.492**
		*P-value*	<.001
HADS	**Anxiety**	*Spearman R*	−.369**
		*P-value*	<.001
	**Depression**	*Spearman R*	−.577**
		*P-value*	<.001
FSS	**Fatigue**	*Spearman R*	−.413**
		*P-value*	<.001

**Correlation is significant at the 0.01 level (2-tailed).

*Correlation is significant at the 0.05 level (2-tailed).

As listed in Table 3, all sections of the UPDRS scale of disease severity had a significant inverse correlation with the total score of the MNA including the mental (Spearman r = −0.503, *P*<0.001), ADL (Spearman r = −0.518, *P*<0.001), motor (Spearman r = −0.473, *P*<0.001) and complication (Spearman r = −0.336, *P*<0.001) parts as well as the total UPDRS score (Spearman r = −0.613, *P*<0.001). Also, the more severe motor symptoms (Hoehn and Yahr: Spearman r = −0.414, *P*<0.001) and morbidity (Schwab and England: Spearman r = 0.492, *P*<0.001) observed, the lower the total MNA scores recorded among the recruited PD patients. All of the evaluated non-motor symptoms were significantly correlated with the MNA scores (all *P*<0.01). However, the HADS score of depression was the strongest one associated with the total score of the MNA questionnaire (Spearman r = −0.577, *P*<0.001). Moreover, both anxiety (Spearman r = −0.369, *P*<0.001) and fatigue (Spearman r = −0.413, *P*<0.001) scores were inversely correlated with the total MNA score. The probable associations of two other continuous variables, age and disease duration, with the MNA score were also assessed. Interestingly, total MNA score was significantly inversely correlated with disease duration (Spearman r = −0.284, *P* = 0.001) in the absence of such an association with patients’ age (*P* = 0.271). In addition, while no correlation was found between the cumulative daily dosage of levodopa and total MNA score (Spearman r = −0.085, *P* = 0.310), the weight-adjusted levodopa dose was inversely correlated with total MNA score (Spearman r = −0.218, *P* = 0.009). The result of the Mann-Whitney *U* test demonstrated that the mean of the total MNA score was significantly lower among the female PD patients compared to the male cases [23.7 (SD = 4.3) vs. 25.8 (SD = 2.6); *P* = 0.002].

### Univariate Comparisons between Patients with Normal versus Abnormal Nutritional Status

As shown in Table 4, the patients with abnormal nutritional status had a significantly longer history of PD compared to those with normal nutritional status [8.2 (SD = 6.9) *yrs* vs. 6.2 (SD = 4.6) *yrs*; t-value = −2.02, *P* = 0.045]. The results of the independent samples *T* test confirmed that except for stigma, the mean score of other domains of the PDQ-39 questionnaire was significantly different between PD patients with different nutritional status (all *P*<0.05). In addition to each part of the UPDRS scale, the mean of the total UPDRS score was significantly higher among the PD patients with abnormal nutritional status [45.9 (SD = 18.0) vs. 26.4 (SD = 13.6); t-value = −6.50, *P*<0.001]. Accordingly, the median score of the Hoehn and Yahr staging was significantly higher in PD patients at risk of malnutrition compared to those with normal nutritional status [2.5 (IQR = 1.0) vs. 2.0 (IQR = 1.5); t-value = −4.55, *P*<0.001]. Based on the Schwab and England ADL score, the PD patients with abnormal nutritional status were also more disabled compared to the subgroup with normal nutrition [71.2 (SD = 19.6) vs. 85.7 (SD = 13.9); t-value = 4.29, *P*<0.001]. In addition, more severe anxiety [8.8 (SD = 5.2) vs. 5.9 (SD = 4.9); t-value = −3.08, *P* = 0.002], depression [9.0 (SD = 4.2) vs. 3.6 (SD = 3.5); t-value = −7.77, *P*<0.001] and fatigue [5.4 (SD = 1.5) vs. 4.2 (SD = 2.0); t-value = −4.00, *P*<0.001] were found in PD patients with abnormal nutritional status.

**Table 4:** Comparison of the mean [standard deviation (SD)] scores of different motor, psychiatric, fatigue and quality of life (PDQ-39) scales between subgroups of Parkinson’s disease (PD) patients regarding nutritional status (MNA).

Scale	Domain	Abnormal nutritional status *(n = 40)*	Normal nutritional status *(n = 106)*	*t-*value	*P*-value
Baseline	**Age** *(yr)*	61.3 (12.3)	61.3 (9.8)	−0.02	.982
	**Disease duration** *(yr)*	8.2 (6.9)	6.2 (4.6)	−2.02	.045*
	**Body mass index (BMI)** *(kg/m^2^)*	23.9 (3.8)	26.6 (4.3)	3.57	.001*
	**Daily levodopa dose**				
	Crude *(mg)*	817 (450)	841 (490)	0.27	.789
	Weight-adjusted *(mg/kg)*	13.55 (8.92)	11.50 (6.94)	−1.46	.146
PDQ39	**Mobility**	45.6 (26.6)	19.4 (20.7)	−5.61	<.001*
	**Activities of daily living (ADL)**	38.4 (28.9)	18.0 (17.9)	−3.97	<.001*
	**Emotional well-being**	41.3 (22.4)	22.1 (20.8)	−4.90	<.001*
	**Stigma**	22.0 (24.4)	21.5 (25.4)	−0.11	.913
	**Social support**	13.0 (17.2)	5.1 (10.9)	−2.51	.016*
	**Cognitive impairment**	25.5 (20.5)	13.5 (16.6)	−3.64	<.001*
	**Communication**	24.6 (23.9)	10.3 (13.6)	−3.56	.001*
	**Bodily discomfort**	32.5 (20.3)	16.4 (19.6)	−4.30	<.001*
Disease Severity	**UPDRS:** Part I-*mental*	3.8 (3.2)	1.3 (1.4)	−4.69	<.001*
	**UPDRS**: Part II-*ADL*	15.9 (7.8)	9.4 (5.8)	−5.17	<.001*
	**UPDRS**: Part III-*motor*	20.6 (10.1)	13.3 (7.4)	−4.18	<.001*
	**UPDRS**: Part IV-*complications*	5.1 (3.4)	2.9 (2.2)	−3.80	<.001*
	*a. Dyskinesia*	1.7 (2.5)	.8 (1.4)	−2.08	.042*
	b. Wearing off	2.3 (1.4)	1.5 (1.2)	−3.06	.003*
	**UPDRS**: Total	45.9 (18.0)	26.4 (13.6)	−6.50	<.001*
	**Hoehn & Yahr stage #**	2.5 (1.0)	2.0 (1.5)	−4.55	<.001*
	**Schwab & England stage** *(%)*	71.2 (19.6)	85.7 (13.9)	4.29	<.001*
HADS	**Anxiety**	8.8 (5.2)	5.9 (4.9)	−3.08	.002*
	**Depression**	9.0 (4.2)	3.6 (3.5)	−7.77	<.001*
FSS	**Fatigue**	5.4 (1.5)	4.2 (2.0)	−4.00	<.001*

# Values are presented as median (IQR).

### Multivariate Analysis

The continuous score of the whole MNA scale was assigned as the outcome of interest in a forward (stepwise) multivariate linear model where the entire regression was statistically significant (R^2^ = 0.539, *P*<0.001, Table 5- model 1). Of interest, with regard to independent variables that remained significant in the regression model, depression (regression coefficient = −0.352, *P*<0.001), total score of the UPDRS scale (regression coefficient = −0.313, *P*<0.001), weight-adjusted daily levodopa dosage (regression coefficient = −0.190, *P* = 0.006) and patients’ sex (regression coefficient = −0.196, *P* = 0.003) were found to be significantly associated with the total MNA score.

**Table 5:** Multivariate regression models to determine the motor and non-motor factors independently related to total score of the MNA questionnaire (model 1) and having abnormal versus normal nutritional status (model 2) in recruited Parkinson’s disease (PD) patients.

Model 1: Linear regression *(Dependent variable: total MNA score)*					
Significant Variables	Unstandardized Coefficients		Standardized Coefficients	*t*	*P-value*
	*B*	*SEM*	*Beta*		
**Depression score**	−.292	.064	−.352	−4.547	<.001
**Total score of UPDRS**	−.064	.016	−.313	−3.903	<.001
**Gender**	−1.525	.503	−.196	−3.032	.003
**Weight-adjusted levodopa dosage**	−.085	.030	−.190	−2.815	.006
**Constant**	30.153	.504	–	59.868	<.001
**Model 2: Binary Logistic regression ** ***(Dependent variable: abnormal vs. normal nutritional status)***					
**Significant Variables**	***B***	***SEM***	***OR (95% CI)***	***Wald***	***P-value***
**Hoehn & Yahr stage**	.865	.322	2.38 (1.26–4.46)	7.232	.007
**Depression score**	.354	.072	1.42 (1.24–1.64)	24.262	<.001
**Constant**	−4.984	.971	–	26.330	<.001

In both regression models, age at the time of diagnosis, sex, weight-adjusted levodopa dosage, the score of the each part of the UPDRS scale, total UPDRS score, Hoehn & Yahr stage, Schwab & England ADL score, anxiety, depression and fatigue scores were entered as the predictor list.

The logistic regression model was also statistically significant (R^2^ = 0.496, *P*<0.001, Table 5- model 2). The forward conditional model revealed that the Hoehn and Yahr stage [OR = 2.4 (95% CI: 1.3–4.5); *P* = 0.007] and depression score [OR = 1.4 (95% CI: 1.2–1.6); *P*<0.001] independently discriminated the PD patients suffering from malnutrition.

## Discussion

In this study, we used MNA to assess nutritional status in a group of PD patients with the mean age of 61 (SD = 10) *yrs,* which is close to the age range of the previous report by Wang et al. [65 (SD = 9) *yr*] who used MNA in the PD population [11]. Although the age distribution of our participants was skewed through the inclusion of elderly individuals, it ranged between 32 and 84 *yr*. As an assessment tool, MNA has shown reliable and valid results to screen those elderly patients at risk of malnutrition [31]; however, there are some reports that reveal reproducible results of using MNA in younger adults aged >18 years [32] or in those with a mean age of around 41 *yrs* [33], though both studies were conducted in patients with chronic kidney disease. Specifically in the case of the PD population, Sheard et al. showed the superior validity of the short form of the MNA compared to other screening tools such as BMI and weight changes. Nevertheless, the Patient-Generated Subjective Global Assessment (PG-SGA) demonstrated better results compared to the full MNA questionnaire for nutritional assessment in a group of PD patients who are aged between 35 and 92 *yrs* [34].

In our study population, malnutrition was found in 2% of the PD patients, while almost a quarter of them were at risk of malnutrition. This figure is close to the reports from the studies by Wang et al. [11] and Barichella et al. [12], illustrating 20% and 22.9% of the risk of malnutrition in Chinese and Italian PD populations, respectively. The rate of malnutrition was also estimated to be as low as 1.7% by Wang et al., which was contributed to race, age, Hoehn and Yahr stage, source of participants’ recruitment (from outpatient clinics) and disease duration [11]. On the other hand, there are some other reports showing more prevalent malnutrition in PD populations to as high as 15.6% [35], 19.5% [36] and 23.6% [37] using BMI measures.

Our findings demonstrate that many motor, psychiatric and fatigue symptoms are significantly associated with nutritional status in PD patients. PD patients with abnormal nutritional status (including malnourished and at risk of malnutrition) had more severe symptoms in all parts of the UPDRS scale including motor, non-motor, ADL and complications. Both dyskinesia and the wearing-off phenomenon were more common among PD patients with abnormal nutritional status in whom the Hoehn and Yahr stage and the Schwab and England ADL scores also showed more severe disease. Among the evaluated psychiatric features, more severe depression, anxiety and fatigue were reported in PD patients with nutritional problems. Besides PD symptoms, health-related QoL was also affected by nutritional status. As our findings show, except for stigma, in all other domains of the PDQ-39, the PD patients with abnormal nutritional status showed worse QoL-scores. In other words, poor nutritional status was accompanied with poor QoL in a group of patients with chronic conditions such as PD. It is noteworthy that different domains of QoL such as disability-related features, cognition, communication, and emotional and social aspects were all correlated with the MNA score in the PD population. To our knowledge, this study is one of the few that directly assess the relationship between nutritional status and HRQoL by means of MNA and PDQ-39 tools in the PD population. However, using MNA in another elderly population, malnourished subjects or those at risk of malnutrition have been shown to have poorer QoL and function in rehabilitation centres [2].

Based on multivariate regression analysis, female gender, higher weight-adjusted daily levodopa dosage, more severe disability (higher UPDRS score, more advanced Hoehn and Yahr stage) and more severe depression predicted a lower MNA score or a higher risk of nutritional deficiency in the PD population, when univariate-independent relationships were adjusted for other covariates. These factors represent the role of demographic (sex), disease severity (UPDRS, Hoehn and Yahr stage), psychiatric (depression) and pharmaceutical (weight-adjusted daily levodopa dosage) features of PD in association with nutritional status. We found significantly lower MNA scores among the female PD population, which is in line with the previously published reports indicating female gender as a risk factor for malnutrition in the elderly based on the total MNA score [38]. With regard to the Hoehn and Yahr stage as a measure of PD severity, the current literature is controversial. Two previous reports have shown no significant difference in the Hoehn and Yahr stage when comparing poor nutritional status with the well-nourished PD patients [11], [39]. In contrast to these previous reports and similar to our current study, Markus et al. [37], Beyer et al. [40], Uc et al. [35] and Sheard et al. [41] reported a statistically significant correlation between Hoehn and Yahr stage and either BMI, the amount of weight change or nutritional status in a PD population.

Regarding psychiatric symptoms and fatigue, there are few similar studies to directly evaluate the relationship between these symptoms and nutritional status in PD. Our findings are in line with previous reports, which show that neuropsychiatric symptoms such as depression, anxiety, dementia, confusion, and apathy contribute to decreased food intake and subsequent weight loss in an elderly PD population [35], [42], [43]. In line with the results of our study, both Wang et al. [11] and Sheard et al. [41] reported depression as a significant predictor of nutritional status in PD patients. Wang et al. [11] considered many non-motor symptoms in their study and showed that both depression and anxiety were associated with a risk of malnutrition in PD. More recently, a higher depression score was shown in malnourished PD patients by using the Beck’s depression inventory (BDI) [41]. Our study uniquely considered fatigue as another factor that might interact with the negative association of depression and anxiety with nutritional status in PD patients. We showed that in addition to previously evaluated psychiatric symptoms, fatigue significantly correlated with malnutrition in PD. Having assessed nutritional status by MNA in another elderly population, Bollwein et al. underlined the close association between frailty syndrome (including exhaustion, low physical activity and slow walking speed) and nutritional status [44]. However, fatigue and exhaustion seem to be both the cause and consequence of malnutrition in the elderly. Emotional well-being, depression and fatigue are important determinants in health status as they are directly associated with the general condition in PD. Additionally, PD can lead to a reduction in mobility, hindering older persons from performing daily activities, such as shopping or food preparation [45].

Our study confirms that more severe motor symptoms and more common dyskinesia are found among PD patients with abnormal nutritional status, which is in line with a similar study performed by Sheard et al. [41]. Although using another scale (SGA) to evaluate nutritional status in a PD population, they also observed higher UPDRS scores among the malnourished participants [41]. The association between malnutrition and disease severity may be due to eating and digesting difficulties occurring in PD [46]. Hypothetically, people with better nutritional status, as indicated by a higher MNA score, may demonstrate a more nutritious dietary pattern when younger, having maintained their healthy diet into late-adulthood. This would increase their odds of being in the groups with higher MNA scores. However, it is not possible to determine the temporality between nutritional status and PD severity by a cross-sectional designed study.

In the present study, BMI was also closely correlated with the nutritional status assessed by MNA. An average of a three-unit lower BMI was observed in PD patients at risk of malnutrition in our study. This finding is in line with a previous report that shows an even lower BMI among the moderately malnourished PD cases compared to the well-nourished patients (20.0 *kg/m^2^* vs. 25.9 *kg/m^2^*) [10]. In parallel, it has been previously shown that different doses of levodopa administered as the main PD treatment may affect BMI levels [46]. In contrast, no association was detected by Nozaki et al. between PD treatment and weight loss [47]. This can be seen in other studies where no correlation was found between levodopa dose, duration, and weight changes [48]. Similarly, we did not find any relationship between the daily dosage of levodopa and nutritional status, and Sheard et al. also reported this lack of association [41]. However, they found a relationship between the weight-adjusted dose of levodopa (*mg* per *kg* body weight) and MNA score [41]. Of interest, we also observed an inverse correlation between the weight-adjusted daily dosage of levodopa and total MNA score, which demonstrates a higher adjusted dose in PD patients with worse nutritional status (lower MNA score). This finding is consistent with the report by Barichella et al. who recently confirmed that nutritional risk depends on weight-adjusted levodopa dose in PD outpatients [49]. These findings highlight an intrinsic association between nutritional status and both the course of disease and levodopa-related complication in PD patients [49], [50], [51].

Most interestingly, an inverse relationship was also found between MNA score and disease duration. While a lower MNA score was accompanied by longer disease duration in PD patients, no association was found between the MNA score and patient’s age. Previously published evidence shows conflicting results on this issue. While no difference in nutritional status, assessed by MNA, was found regarding PD duration in one study [11], Barichella et al. demonstrated a linear correlation between the MNA score and the duration of PD, even though no correlation was observed between MNA and the chronological age of the patients [12]. These patterns are quite consistent with our findings in the Iranian PD population, which showed an independent association between MNA and PD duration.

Our study suffers from some limitations, which must be taken into account when generalising its findings. One issue refers to the fact that no serum measurement was performed to quantify blood indicators of nutrition. Similar to most previous reports on this issue, the primary focus is on BMI and weight loss, whereas other indicators of nutritional condition such as food intake, fat and muscle status are not considered in our study. However, as an innovative clinical evaluation instrument, the MNA questionnaire provides several questions and four anthropometric measurements without any need for blood sampling or other clinical measurements. MNA is an acceptable tool to assess nutritional status especially among those elderly who are aged >65 *yrs*; therefore, our findings regarding younger patients might not be as valid as those of older ones. Nevertheless, this is the real-life condition of PD population in referral movement disorder centres, and other reports with rather similar ranges of age also used this tool and showed its validity in PD [11], [34]. Our study has primarily been designed as a cross-sectional study, which did not allow for the determination of directionality of the association or the so-called causality between malnutrition and PD-related factors. Similar to previous studies, it is not clear whether poor nutritional status is “the chicken or the egg”, i.e. the cause or the effect [52]. In a cross-sectional study, such as ours, the relationship between PD symptoms and nutritional status can be reciprocal. While poor symptom control can increase the risk of poor nutritional status, malnutrition can also result in poor symptom control. Moreover, the recruited patients in our study were PD outpatients who attended a referral movement disorder clinic. This selection bias has restricted our samples to the group of mild-to-moderate stage non-hospitalised PD patients. The more severe or end-stage cases, which are potentially at a higher risk of malnutrition, were not able to participate in this study. In order to have more valid data, patients with moderate to severe dementia were also excluded. However, as dementia is a known risk factor of poor nutritional status, this exclusion criterion can also further restrict the generalisability of our findings. As a result, our findings are better to be generalised into an outpatient PD population with mild-to-moderate stages of the disease who are not suffering from severe dementia. Finally, as has been observed in other clinical recordings, some parts of the information were self-reported and based on interviews with the patients and/or their caregivers, hence liable to inaccuracies. Recall bias may have also occurred, causing over- or under-estimated reports for some of the answers. Besides the limitations, our study is the first investigation to contribute to a better understanding of the nutritional status among Iranian PD patients. This study benefits from a large sample size, leading to an acceptable statistical power, as well as a comprehensive list of motor and psychiatric symptoms and the HRQoL, which makes the findings reliable and precise. In addition, we used the MNA questionnaire as an accepted tool to assess nutritional status, while many of the previous studies commonly used BMI and/or body weight instead.

In conclusion, evaluation of nutritional status using the MNA questionnaire in this population of PD patients has demonstrated that there is a significant correlation between PD duration and nutritional status. This significant correlation without considering chronological age, reduces the possibility that the worse MNA scores in patients with longer PD duration were due only to increasing age. In addition, we indicated that PD-related factors are associated with malnutrition in these patients. Many non-motor psychological aspects of PD were related to nutritional status as well as disease severity and level of morbidity. Different aspects of the HRQoL were also closely associated with patients’ nutritional status. These cross-sectional data raise further questions and hypotheses regarding whether the individuals with insufficient nutrient intake in the past are more likely to experience malnutrition and a more severe course of PD, or if the presence of PD symptoms can hinder the capability or willingness to engage in appropriate nutritious meal preparation.

The significant correlations between the non-motor and motor symptoms of PD with malnutrition and its association with QoL bring to light the idea of using a tool to assess nutritional status in the routine evaluation of PD as the disease progresses. MNA appears to be a practical, user-friendly and cheap assessment tool for this purpose. Although the role of nutrition in the progression of neurodegenerative disorders still needs more clarification, theoretically, a vicious circle can be created if nutritional features of PD are ignored that can themselves worsen their QoL. Nevertheless, conducting follow-up longitudinal assessments and further studies involving PD patients with more severe stages of the disease may help in providing a more comprehensive picture of the nutritional status and the inter-relationships with motor and psychiatric symptoms and fatigue in PD patients.

Overall, nutritional screening using instruments such as MNA is an easy and quick way to identify patients with PD who may be at risk of malnutrition. They can then be assessed to determine if they are malnourished and to determine appropriate interventions. Based on our findings, PD patients with more depressive symptoms, severe fatigue and more severe disability who are under higher weight-adjusted levodopa dose are more likely to suffer from poor nutritional status and may benefit more from nutritional screening and follow-up assessment. Regardless of the direction of this relationship, including dietary education in the care approaches used for PD patients seems to improve their QoL.
